# Myocardial Infarction Following COVID-19 Vaccine Administration: *Post Hoc, Ergo Propter Hoc*?

**DOI:** 10.3390/v14081644

**Published:** 2022-07-27

**Authors:** Arianna Baronti, Francesco Gentile, Alice Chiara Manetti, Andrea Scatena, Silvia Pellegrini, Angela Pucci, Maria Franzini, Vincenzo Castiglione, Aniello Maiese, Alberto Giannoni, Mauro Pistello, Michele Emdin, Giovanni Donato Aquaro, Marco Di Paolo

**Affiliations:** 1 Institute of Legal Medicine, Department of Surgical, Medical and Molecular Pathology and Critical Care Medicine, University of Pisa, 56126 Pisa, Italy; a.baronti4@studenti.unipi.it (A.B.); a.manetti3@studenti.unipi.it (A.C.M.); a.scatena2@studenti.unipi.it (A.S.); aniello.maiese@unipi.it (A.M.); marco.dipaolo@unipi.it (M.D.P.); 2 Fondazione Toscana G. Monasterio, 56124 Pisa, Italy; frage1993@gmail.com (F.G.); vincenzocastiglione93@gmail.com (V.C.); alberto.giannoni@gmail.com (A.G.); aquaro@ftgm.it (G.D.A.); 3 Department of Clinical and Experimental Medicine, University of Pisa, 56126 Pisa, Italy; silvia.pellegrini@unipi.it; 4 Department of Histopathology, University of Pisa, 56126 Pisa, Italy; angelapucci@libero.it; 5 Department of Translational Research and of New Surgical and Medical Technologies, University of Pisa, 56126 Pisa, Italy; maria.franzini@unipi.it; 6 Health Science Interdisciplinary Center, Scuola Superiore Sant’Anna, 56127 Pisa, Italy; 7 Retrovirus Center and Virology Section, Department of Translational Research, University of Pisa, 56126 Pisa, Italy; mauro.pistello@unipi.it

**Keywords:** COVID-19, vaccine, myocardial infarction

## Abstract

Vaccination against coronavirus disease 2019 (COVID-19) is the safest and most effective strategy for controlling the pandemic. However, some cases of acute cardiac events following vaccine administration have been reported, including myocarditis and myocardial infarction (MI). While post-vaccine myocarditis has been widely discussed, information about post-vaccine MI is scarce and heterogenous, often lacking in histopathological and pathophysiological details. We hereby present five cases (four men, mean age 64 years, range 50–76) of sudden death secondary to MI and tightly temporally related to COVID-19 vaccination. In each case, comprehensive macro- and microscopic pathological analyses were performed, including *post-mortem* cardiac magnetic resonance, to ascertain the cause of death. To investigate the pathophysiological determinants of MI, toxicological and tryptase analyses were performed, yielding negative results, while the absence of anti-platelet factor 4 antibodies ruled out vaccine-induced thrombotic thrombocytopenia. Finally, genetic testing disclosed that all subjects were carriers of at least one pro-thrombotic mutation. Although the presented cases do not allow us to establish any causative relation, they should foster further research to investigate the possible link between COVID-19 vaccination, pro-thrombotic genotypes, and acute cardiovascular events.

## 1. Introduction

The Severe Acute Respiratory Syndrome CoronaVirus-2 (SARS-CoV-2) pandemic and its related disease (COVID-19) have fostered strenuous efforts toward finding an effective preventive strategy [1,2]. Following the encouraging results of randomized controlled trials (RCTs) [3], the mRNA vaccine BNT162b2 (Comirnaty^®^, BioNTech/Pfizer, Mainz, Germany), followed by mRNA1273 (Spikevax^®^, Moderna, Cambridge, MA, USA), Ad26.COV2.S (Janssen^®^, Johnson&Johnson, New Brunswick, NJ, USA), and ChAdOx1 nCoV-19/AZD1222 (Vaxzevria^®^, AstraZeneca, Cambridge, UK), received emergency approval from international regulatory agencies and were administered on a global scale. More recently, NVX-CoV2373, a vaccine made up of spike protein trimers assembled into nanoparticles (Novavax^®^, Novavax Inc., Gaithersburg, MD, USA), has been approved for human use. Over the last 2 years, vaccination has been confirmed as the safest and most effective strategy for containing the pandemic [3,4], and as of May 2022, about 10 billion doses were administered worldwide [5,6,7]. Nevertheless, considering the preventive nature of vaccines, tight control of their safety profile over time is necessary. Since even large RCTs might be underpowered to detect rare adverse events (AEs), the vaccination campaign is continuously monitored to report any AE; establish their severity; and, whenever possible, assess causality relations as part of the safety surveillance or pharmacovigilance campaign [8,9].

Various case reports or case series have already reported acute cardiovascular events of either an inflammatory (e.g., myocarditis) or thromboembolic nature (e.g., venous thromboembolism, stroke, or myocardial infarction—MI) and have been potentially associated, at least temporally, to COVID-19 vaccines [10]. Although the precise number of post-vaccination MI is difficult to retrieve, a recent systematic review reported 35 non-fatal MI cases (28 men, mean age 65 years, range 59–74) [10].

Vaccine-induced thrombotic thrombocytopenia (VITT) has been identified as a possible mechanism [11], but, the pathogenic *noxa* underlying most cases remains undetermined [10]. Furthermore, considering the high incidence of MI in the general population, a temporal correlation with the vaccination is not sufficient to establish a causal relationship [12]. Since a more comprehensive autoptic evaluation of lethal cases may provide crucial pathophysiological details [13,14,15,16], we analyzed all of the cases of fatal MI temporally related to COVID-19 vaccination referred to our institute, complementing the macroscopic and microscopic examinations with *post-mortem* cardiac magnetic resonance (PM-CMR) and genetic tests. Furthermore, considering that some specific genotypes may be predisposed to thrombotic events in the presence of various triggers, we investigated the prevalence of these polymorphisms in the study subjects.

## 2. Methods

### 2.1. Inclusion Criteria

Since the beginning of the National vaccination campaign in Italy (January 2020 to March 2022), all cases of sudden cardiac death that were referred for a judicial autopsy at the Institute of Legal Medicine of Pisa (Italy) and the cases that were temporally related to the administration of a COVID-19 vaccine were considered for this study.

### 2.2. Exclusion Criteria

An infection from SARS-CoV-2 at the time of death, extracardiac causes of sudden death (e.g., pulmonary, cerebral), and the presence of putrefactive phenomena were considered as exclusion criteria. Therefore, before autopsy, a nasopharyngeal swab was obtained in order to detect SARS-CoV-2 and then analyzed according to the current recommendations [17]. In the case of a sudden cardiac death, all autoptic studies were performed within 24 h from death, in accordance with the guidelines of the Association for European Cardiovascular Pathology, [18], and a sequential approach was performed to exclude other extra-cardiac causes of sudden death.

### 2.3. Post-Mortem Cardiac Magnetic Resonance

In the case of an absence of signs of MI during the external inspection of the heart, a PM-CMR was performed, before proceeding with further histopathological analyses. All of the exams were performed 7 days after heart fixation using a 1.5-Tesla MR scanner (Signa HD CVi and Signa Artist, GE Healthcare, Chicago, IL, USA) with a multichannel cardiac phased array coil, as previously described [19,20]. The acquisition protocol included a whole-heart 3D steady state free precession (SSFP) pulse sequence, T1- and T2-weighted sequences, and T1 and T2 mapping. Dedicated post-processing software (CVI-42 Circle) was used to analyze the acquired images [21].

### 2.4. Histopathological and Immunohistochemical Analysis

After the PM-CMR, the cardiac tissues were examined according to established protocols [18]. The epicardial coronary arteries were serially cut (after the decalcification of the calcified vessels) at 5 mm intervals and visually examined. After the external examination and inspection, each heart was cut along the short axis from the apex to the mid-ventricular level, at 10 mm-thick cuts, and each slice was visually inspected to evaluate any macroscopic change. All of the specimens were formalin-fixed and paraffin-embedded (FFPE), stained with hematoxylin–eosin (H&E), and microscopically analyzed by using a Leica DM4000B optical microscope (Leica Microsystems).

In the cases with a suspicion of MI during the PM-CMR but lacking any macroscopic signs, an immunohistochemical analysis was also performed. Briefly, the serial 3 mm-thick FFPE heart sections were mounted on slices covered with 3-aminopropyl-triethoxysilane (Fluka Chemie AG), and single immunostainings were performed by using specific antisera raised against anti-gap junction proteins, including Connexin-43 (CX43 polyclonal antibody, Elabscience cat. No. E-AB-30999) and non-phosphorylated (np) Cx43 (monoclonal antibody, Thermo Fisher Scientific, Cat. No. 13-8300), as previously described [22]. The reactions were revealed by the immunoperoxidase technique with a 3′-5′-diaminobenzidine chromogen substrate. The stained slides were blindly and independently evaluated by 2 expert examiners (AP and MDP), who considered an increased npCx43/Cx43 expression ratio in the cytoplasm and in the intercalated discs to be diagnostic of MI, concomitant to the decreased Cx43 immunoreactivity, as previously validated [22].

### 2.5. Toxicological Analysis

A comprehensive toxicological examination using liquid chromatography–high resolution mass spectrometry (LC-HRMS) was performed on venous blood samples, screening for ethanol, cocaine, benzodiazepines, ketamine, norketamine, opioids, methadone, amphetamine, meta-amphetamines, and cannabinoids.

### 2.6. Antibodies Anti-Platelet Factor 4 and Tryptase Analysis

The presence of the anti-platelet factor 4 (anti-PF4) antibodies and the tryptase serum concentration were evaluated to rule out the diagnosis of VITT and anaphylaxis. Serum samples that were obtained through a venous blood centrifugation (10,000× *g*, 10′, room temperature) were screened for the presence of anti-PF4/polyanion complex antibodies, using the commercially available PF4 IgG test (Immucor GTI Diagnostics). The tryptase concentration was determined by the ImmuoCAP assay using the automated Phadia 250 instrument (Thermo Fisher Scientific).

### 2.7. Genetic Analysis

Whole blood samples collected in ethylenediaminetetraacetic acid tubes were used for the genomic DNA extraction and genotyping and executed using the CE-In Vitro Diagnostic (IVD) validated ELITe InGenius instrument (EliTech Group, Puteaux, France). In particular, (a) Factor V G1691A (Leiden), Factor II G20210A, and methylenetetrahydrofolate reductase (MTHFR) C677T genotypes were assessed by the Coagulation ELITe MGB kit (EliTech Group, Puteaux, France), i.e., a real-time polymerase chain reaction assay designed to discriminate the allelic variants by analyzing the amplicon melt-curves; (b) MTHFR A1298C and Factor V A4070G (FV-HR2) were genotyped using the quantitative-PCR Alert AmpliMIX (EliTech Group) and an allele-specific fluorescent probe hybridization; (c) Factor XIII V34L (g.6318795C > A), HPA-1 a/b (GPIIIa g.1565T > C), plasminogen activator inhibitor-1 (PAI-1) 4G/5G (g.101126425_101126426insG), and Beta-Fibrinogen 455G > A were genotyped using a Duplic α Real-time genotyping kit (Euroclone), and an allele-specific fluorescent probe hybridization. While the pro-thrombotic role of some of these polymorphisms has been widely confirmed (e.g., for Factor II G20210A and Factor V G1691A), it is less clear for others, such as the MTHFR variants, whose clinical significance is proportional to the related hyperhomocysteinemia [23].

## 3. Results—Case Description

Finally, a total of five cases of sudden cardiac death temporally related to the administration of a COVID-19 vaccine were referred to our institute for autopsy between February 2021 and December 2021. Since all of the nasopharyngeal swabs were negative for SARS-CoV-2 and neither extracardiac causes of sudden deaths nor putrefactive phenomena were detected, all of the subjects were enrolled in the study. A brief description of the cases (four men and one woman, mean age 64 years, range 50–76) is provided in Table 1 and Table 2. Three subjects had previously known medical conditions, including diabetes mellitus (#1), chronic obstructive pulmonary disease (#1), hypertension (#4), and cocaine abuse history (#5).

In all of the cases, mRNA vaccines were administered: the BNT162b2 vaccine in four cases, as either the first (*n* = 2, #1 and #3) or the second (*n* = 2, #2 and #4) dose, and a first dose of mRNA1273 in the remaining case (#5). The vaccination-to-death interval was <24 h in two cases (#2 and #5), 24–72 h in two other cases (#1 and #4), and 21 days in the last case (#3). In three cases (#1, #2, and #4), death was unwitnessed, although in the preceding hours, the deceased had reported symptoms compatible with acute myocardial ischemia (e.g., thoracic pain) to family members. In the other two cases (#3 and #5), the circumstances of death and symptoms remained unknown.

**Figure 1:**
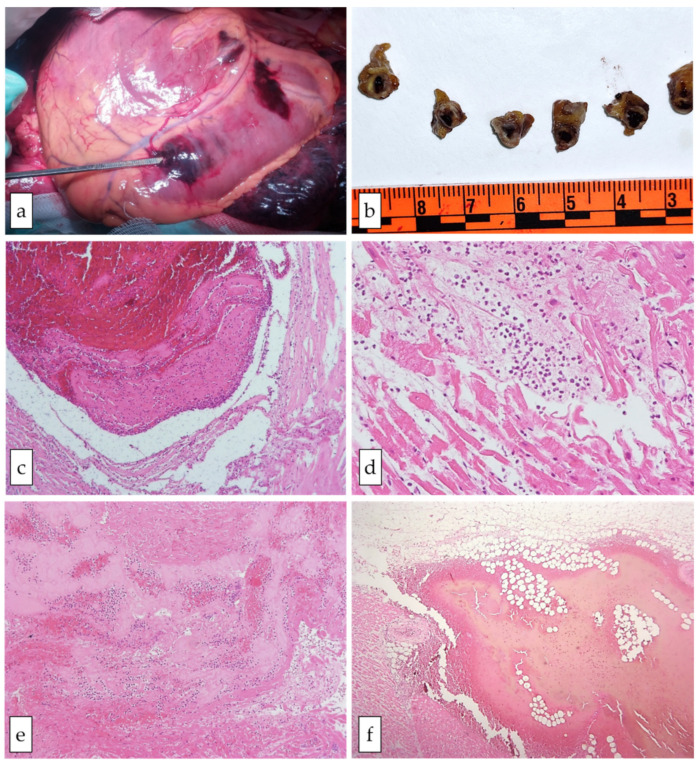
**Gross and microscopic cardiac features in case #1.** Panel (**a**) shows the transmural myocardial rupture. Panel (**b**) shows the transversal section of the coronary artery with evidence of atherosclerotic changes and an intracoronary clot. In panels (**c**–**f**), the histological findings of the myocardial sections are presented (hematoxylin–eosin staining): a fresh thrombus in the coronary artery (**c**, original magnification 10×); inflammatory infiltrates (neutrophils) and myocyte contraction band necrosis (**d**, original magnification 20×); myocardium adjacent to the rupture site with intense inflammatory infiltrates, fibrin, and coagulative necrosis (**e**, original magnification 10×); hemorrhage in the epicardial adipose tissue close to the left ventricular (LV) free wall rupture (**f**, original magnification 20×).

**Figure 2:**
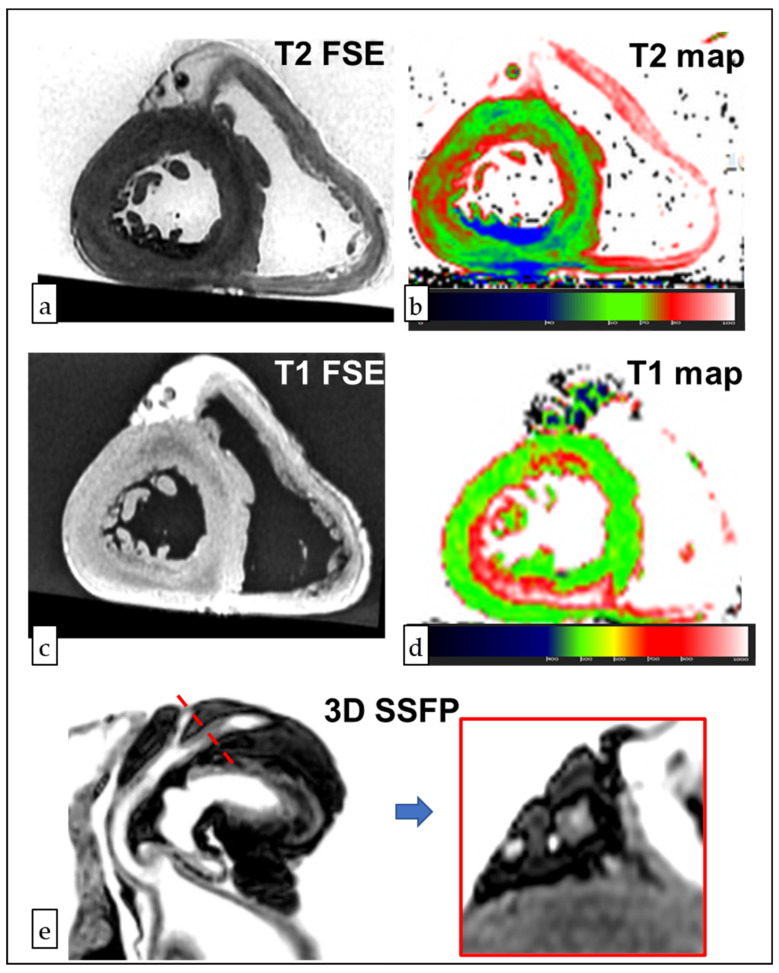
***Post-mortem* Cardiac Magnetic Resonance (PM-CMR) and immunohistochemical staining of case #4.** In the panel (**a**), a T2-weighted fast spin echo (FSE) image shows the hypointensity of the subendocardial layer of the inferior and inferolateral walls. A decreased T2 was confirmed in the T2 mapping (panel **b**), whereas T1 hyperintensity and an increased T1 were found in the border zone (T1 FSE of panel **c**, T1 mapping in panel **d**). The reconstruction of 3D-SSFP images showed a >90% obstruction of the left circumflex artery (panel **e**).

While the macroscopic signs of MI were observed in two cases (#1 and #3), characterized by myocardial wall rupture and hemopericardium in the posterior wall of the left ventricle in both cases (Figure 1), no gross alterations were observed in the other cases (#2, #4, and #5). A PM-CMR was therefore performed in these cases. The PM-CMR showed ischemic lesions in all of the hearts that presented with signs of hyperacute myocardial ischemia (subendocardial hypointensity in T2-FSE and low T2 at mapping) and with surrounding oedema (higher myocardial T1 in the border zone). Moreover, the 3D-SSFP sequence reconstruction unveiled obstructive or sub-obstructive coronary artery stenosis in each case (Figure 2). Such findings were confirmed in the macroscopic examination of the coronary arteries, showing the three-vessel disease in all of the cases, with a fresh thrombus in four individuals (#1, #2, #4, and #5), involving either the left anterior descending artery (#2, #4, and #5) or the right coronary artery (#1).

**Figure 3:**
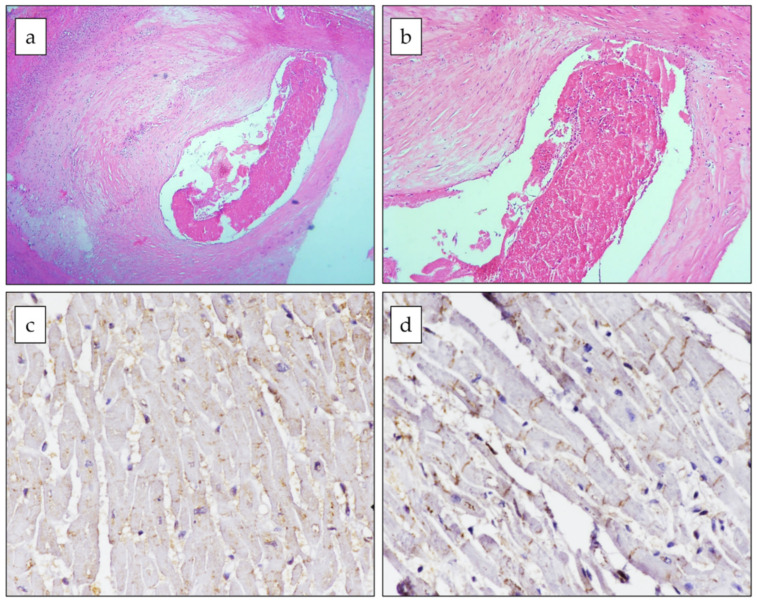
**Histological and immunohistochemical features of case #4.** A fresh sub-occlusive thrombus (**a**,**b**) is shown in the lumen of the right coronary artery with fibroatheroma (hematoxylin–eosin staining, original magnification 4× and 10×, respectively); immunohistochemistry (**c**,**d**) showed phosphorylated (CX-43) and dephosphorylated connexin-43 (npCX-43) expressions in the ischemic myocardium (original magnification 40×). CX-43 was mainly localized to the cytoplasm (**c**), whereas npCX43 was mainly expressed at the intercalated disk and almost absent in the cytoplasm (**d**).

Through the microscopic examinations, findings of early MI were detected in four cases (#1, #2, #4, and #5), in which the only histological changes were signs of stretching and waviness of the fibers, while in case #3, the histology was compatible with a 15/20-day-old MI, showing some macrophage infiltration of the necrotic infarcted area and granulation of the tissue at its margins, as previously described [24]. Furthermore, in cases #2, #4, and #5, findings suggesting acute ischemia were documented through an immunohistochemical analysis (Figure 3).

Finally, the toxicological screening, anti-PF4 antibodies, and tryptase analyses yielded negative results in all of the cases. Through genetic testing, all of the subjects were identified as carriers of ≥1 MTHFR pro-thrombotic allele, and four individuals (#1, #2, #3, and #4) were also carriers of one or two PAI-1 4G alleles (Table 3).

## 4. Discussion

In this original series, we presented the pathological features of five cases of sudden death temporally related to the administration of a vaccination against COVID-19 and referred to the Institute of Legal Medicine of Pisa for *post-mortem* investigations. To ascertain the cause of death of these subjects, a comprehensive autoptic study was conducted, complemented by radiological (PM-CMR), histological, immunohistochemical, toxicological, and genetic investigations. Notably, MI was identified as the cause of death, and a consistent temporal relationship between the MI occurrence and the vaccine administration was observed in all cases. A cardiac rupture with tamponade was the cause of death in two cases, whereas non-mechanical MI complications could have been the final cause of death in the other cases.

Although coronary artery lesions of variable severity were found in all of the cases, none of the subjects had a known previous history of cardiovascular disease, except for the presence of some common risk factors. The toxicological screening as well as the tryptase quantification were negative in all cases. While the absence of anti-PF4 antibodies virtually ruled out the diagnosis of VITT, and genetic testing revealed that all of the subjects were carriers of at least one pro-thrombotic mutation.

In the last 2 years, several RCTs and observational studies have confirmed that a global vaccination against COVID-19 is the most effective and safest strategy in order to contain the pandemic [25,26,27,28]. AEs after a COVID-19 vaccination are very rare. However, considering their preventive nature, the safety of vaccines is paramount, and the International Regulatory Agencies recommend tight control of any possible AEs, and the assessment of their epidemiological and clinical characteristics. Indeed, the vaccination campaign is still ongoing and almost certainly will be prolonged over time. Therefore, the identification of subjects at risk of severe AEs could be crucial in order to reassess the risk–benefit ratio of the vaccination in specific circumstances.

Unfortunately, establishing a causal link between an AE and anti-COVID-19 vaccination may not be easy, particularly in the case of relatively frequent conditions such as cardiovascular events [29]. Therefore, although the suggestive temporal correlation, no definitive causal relationship with vaccine administration could be postulated in the subjects analyzed in this series.

To date, various studies have reported a possible association between cardiovascular events and COVID-19 vaccination [10,30], unveiling a pathophysiological link in the case of VITT [11,31,32,33,34]. Accordingly, among the cases of MI temporally related to vaccine administration [15,35,36,37,38,39,40], VITT was identified as the possible underlying pathogenic mechanism in one subject [41]. Of note, among the five subjects analyzed, testing for anti-PF4 antibodies was negative in all cases, suggesting that VITT was not implicated. Similarly to our observations, Tajstra et al. described a case of fatal MI secondary to the multivessel coronary thrombosis in an 86-year-old man 30 min after vaccine administration, though no pathophysiological mechanisms were identified [36]. On the other hand, in a case series including three subjects who presented with cardiac-related manifestations shortly after the COVID-19 vaccination, the final diagnosis was different in each patient and included acute MI, stress-induced cardiomyopathy, and MI with nonobstructive coronary arteries [38]. Therefore, other possible mechanisms have been proposed to explain the potential relationship between MI and COVID-19 vaccination. Among these, a disproportionate stress-related adrenergic reaction might contribute to a coronary spasm, plaque erosion, and acute thrombosis [38]. Similarly, an intense inflammatory reaction to the vaccines might be involved. Notably, Kounis syndrome has been historically described as the occurrence of an acute coronary syndrome following either vaccination or exposure to other allergens [42]. Although the precise mechanisms have yet to be clarified, hypersensitivity, cellular, and biohumoral mediators might promote a coronary spasm, plaque erosion, and pro-thrombotic cascades [43]. Although in the presented cases, negative tryptase testing virtually ruled out allergic reactions, it could not be excluded that the delayed sample collection might have yielded some false-negative results secondary to the *post-mortem* degradation, as reported in up to 20% of cases of Kounis syndrome [44].

Contrary to the case reports published so far, we also performed a comprehensive genetic analysis in order to identify the pro-thrombotic genotypes. Interestingly, although in the absence of any previous history of thromboembolic events, all subjects were carriers of at least one pro-thrombotic allele of the MTHFR gene. Specifically, two subjects were heterozygotes for both MTHFR C677T and A1298C, one subject was homozygous for MTHFR A1298C-C, one subject was heterozygous for MTHFR C677T, and one subject was heterozygous for MTHFR A1298C. The MTHFR enzyme converts 5,10-methylenetetrahydrofolate into 5-methyltetrahydrofolate, producing a methyl donor for the re-methylation of homocysteine into methionine. The C677T and the A1289C polymorphisms produce an alanine into valine and a glutamate into an alanine substitution in the MTHFR enzyme, respectively, both of which may blunt enzymatic activity, thereby predisposing them to thrombotic events [45,46]. Interestingly, it has been reported that these polymorphisms might be associated with a greater severity and mortality consequent to COVID-19 [47,48].

Beyond MTHFR, four of the studied subjects were also carriers of one or two 4G alleles of the PAI-1. Briefly, PAI-1 is a serine protease involved in fibrinolysis by inhibiting the tissue plasminogen activator. The deletion/insertion of polymorphisms with four (4G) or five (5G) guanosine residues have been associated with an increased mRNA transcription and PAI-1 concentration, thus reducing fibrinolysis and increasing the risk of venous thromboembolism [49].

Whether the coexistence of both MTHFR and PAI-1 pro-thrombotic polymorphisms might have contributed to the risk of MI in the studied subjects remains unknown, although such an incremental risk has been described for thrombotic events in other settings [50,51]. 

However, it should be recognized that none of the subjects were carriers for any mutations definitively associated with an increased pro-thrombotic risk (such as Factor II G20210A and Factor V G1691A) [23]. On the other hand, the genetic variants identified are not rare in the general population: the double heterozygosity (677 TC/1298 AC) of MTHFR has a frequency of about 34%, while the 1289 CC genotype is present in 9.4% of subjects [52], whose pro-thrombotic risk depends on the presence and severity of hyperhomocysteinemia [23]. Similarly, the PAI-1 4G/4G genotype has a frequency of 29%, while the 4G/5G genotype is present in up to 50% of subjects, being only slightly more prevalent among individuals suffering from cerebrovascular events [52].

Considering these observations, despite the suggestive temporal relation and the possible predisposing pro-thrombotic genotypes of the studied subjects, a direct role of COVID-19 vaccination as a trigger for MI occurrence could not be extrapolated. The reported findings should therefore be interpreted with caution but may stimulate future research on the potential pathophysiological association between the COVID-19 vaccination, pro-thrombotic genotypes, and cardiovascular events.

## 5. Conclusions

Herein, we presented five cases of fatal MI temporally related to the COVID-19 vaccination. Given the limited sample size, these findings do not support a causal relationship between the vaccine administration and the risk of MI. However, considering the ongoing worldwide vaccination campaign, identifying any possible vaccine-related AE is of paramount importance. Adopting a standardized and comprehensive methodology when analyzing and describing these cases is therefore warranted. Notably, in the reported cases, macroscopic, histopathological, immunohistochemical, toxicological, and biohumoral tests did not provide any pathophysiological detail regarding the possible mechanisms leading to the fatal event, whereas the genetic analysis revealed the presence of pro-thrombotic genotypes in all subjects.

## Figures and Tables

**Table 1:** Clinical characteristics and mode of death.

Case Number	Sex	Age, Years	Previous Conditions	Vaccine	Vaccination-to-Death Interval	Circumstances of Death and Previous Symptoms
#1	M	69	DM2, COPD, smoking	BNT162b2, 1st dose	48 h	Unwitnessed Complained of shivering, thoracic, and upper limbs pain some hours before
#2	M	58	None	BNT162b2, 2nd dose	~8 h	Unwitnessed Complained of thoracic pain < 1 h before
#3	M	76	None	BNT162b2, 1st dose	21 days	Unwitnessed
#4	M	68	Hypertension	BNT162b2, 2nd dose	72 h	Sudden death during cycling, preceded by headache and thoracic pain
#5	F	50	Cocaine abuse, smoking	mRNA1273, 1st dose	<24 h	Unwitnessed

COPD: chronic obstructive pulmonary disease; DM2, type 2 diabetes mellitus.

**Table 2:** Autoptic, radiological, histological, and laboratory findings.

Case Number	Macroscopic Findings	PM-CMR Findings	Histological and IHC Findings	MI Age	Toxicological Screening	Anti-PF4	Tryptase
#1	Hemopericardium, heart laceration on the posterior wall of the left ventricle, pre-existing critical three-vessel atherosclerotic disease, coronary thrombosis	Not performed	Coronary thrombosis of right coronary artery with significant stenosis. MI at the rupture site.	24–48 h	Negative	Negative	Negative
#2	Pre-existing three-vessel atherosclerotic disease, coronary thrombosis, hypoplastic right coronary artery	Ischemic damage	Coronary thrombosis of left anterior descending artery. IHC diagnostic of MI.	<24 h	Negative	Negative	Negative
#3	Hemopericardium, heart laceration posterior wall of the left ventricle, pre-existing three-vessel atherosclerotic disease	Not performed	MI at the rupture site.	15/20 days	Negative	Negative	Negative
#4	Pre-existing three-vessel atherosclerotic disease, coronary thrombosis	Ischemic damage	Coronary thrombosis of left anterior descending artery. IHC diagnostic of MI.	<24 h	Negative	Negative	Negative
#5	Pre-existing three-vessel atherosclerotic disease, coronary thrombosis	Ischemic damage	Coronary thrombosis of left anterior descending artery. IHC diagnostic of MI.	<24 h	Negative	Negative	Negative

Anti-PF4: anti-platelet factor 4; IHC: immunohistochemistry; MI: myocardial infarction; PM-CMR: post-mortem cardiac magnetic resonance.

**Table 3:** Results of the genetic analysis performed on whole blood samples.

Gene Variant	Case #1	Case #2	Case #3	Case #4	Case #5
*MTHFR C677T*	CT	CT	CC	CT	CC
*MTHFR A1298C*	AC	AC	CC	AA	AC
*Factor II G20210A*	GG	GG	GG	GG	GG
*Factor V G1691A*	GG	GG	GG	GG	GG
*Factor V-HR2*	AG	AA	AA	AA	AA
*Factor XIII V34L*	CA	CA	CA	CC	CA
*HPA-1 a/b*	TC	TC	TT	TT	TT
*PAI-1 4G/5G*	4G/4G	4G/5G	4G/4G	4G/5G	5G/5G
*Beta-Fibrinogen 455G > A*	GG	GG	GG	GA	GG

HPA: human platelet antigen; MTHFR: methylene-tetrahydrofolate reductase; PAI: plasminogen activator inhibitor.
